# Editorial: Unraveling the biological mechanisms of chronic fatigue: a multifaceted approach

**DOI:** 10.3389/fmed.2026.1936679

**Published:** 2026-07-29

**Authors:** Abbas F. Almulla, Benjamin H. Natelson, Leonard H. Calabrese, Fereshteh Jahanbani

**Affiliations:** 1 Sichuan Provincial Center for Mental Health, Sichuan Provincial People's Hospital, School of Medicine, University of Electronic Science and Technology of China, Chengdu, China; 2 Key Laboratory of Psychosomatic Medicine, Chinese Academy of Medical Sciences, Chengdu, China; 3 Medical Laboratory Technology Department, College of Medical Technology, The Islamic University, Najaf, Iraq; 4 Icahn School of Medicine at Mount Sinai, New York, NY, United States; 5 Cleveland Clinic, Cleveland, OH, United States; 6 Center for Complex Chronic Multisystem Conditions (4C), Stanford University, Stanford, CA, United States; 7 Division of Immunology and Rheumatology, Department of Medicine, Stanford University School of Medicine, Stanford, CA, United States

**Keywords:** autonomic dysfunction, biomarkers, long COVID, myalgic encephalomyelitis/chronic fatigue syndrome, neuroimaging, post-viral fatigue, precision medicine

Chronic fatigue, and myalgic encephalomyelitis/chronic fatigue syndrome (ME/CFS) in particular, remain without a validated diagnostic marker or a treatment. The post-COVID rise in post-viral fatigue has further widened the gap between clinical need and mechanistic understanding. This Research Topic brings together studies that examine chronic fatigue across several biological levels, including ion-channel function, skeletal muscle physiology, brain structure and function, autonomic and ventilatory regulation, longitudinal outcome, and potential treatment. The Research Topic does not point to one single mechanism. Its value lies in replacing broad clinical description with measurable biological and functional signals, while identifying several recurring themes across different methods and patient groups.

One important theme concerns ion transport and calcium regulation. Sasso et al. provide a reproducible cellular finding by using whole-cell patch-clamp recordings in natural killer cells across two independent laboratories. They report reduced TRPM3 channel activity in individuals with ME/CFS, with no significant laboratory site effect. Given the role of TRPM3 in calcium signaling, these findings support altered ion-channel regulation as a potential biological feature of ME/CFS. Because TRPM3 was assessed in natural killer cells, this work also connects ion-channel dysfunction with an immune-cell model that has been repeatedly studied in ME/CFS. However, TRPM3 remains a research assay rather than a clinically deployable diagnostic test, and its specificity against other fatiguing or inflammatory conditions still needs to be established.

Wirth and Steinacker approach the problem from a different anatomical level. Their hypothesis and theory article proposes that impaired skeletal-muscle Na^+^/K^+^-ATPase activity may contribute to sarcolemmal depolarization, reduced force generation, and hyperexcitability in severely ill ME/CFS patients. In their model, intracellular sodium loading may promote reverse-mode Na^+^/Ca^2+^ exchange, calcium overload, mitochondrial injury, and worsening energy failure. This model remains theoretical, but it offers a coherent explanation for muscle weakness, fatigue, cramps, and fasciculations in severe disease. Together, the Sasso et al. and Wirth and Steinacker contributions highlight ion transport and calcium regulation as plausible biological pathways, although they examine different cell types and levels of pathology. Given that channelopathy has been proposed as a potential disease mechanism, further research in this area is warranted.

A second theme concerns central nervous system involvement. Kumar et al. examined sensory abnormalities in ME/CFS using event-related potentials. They found no significant difference in early sensory gating, measured by P50 suppression, but observed a reduced P300 response in ME/CFS patients. This pattern suggests that sensory problems in ME/CFS may involve altered higher-order cognitive processing rather than a primary abnormality in early sensory filtering. This interpretation is important because it shifts attention from the sensory organs themselves toward brain systems involved in attention, evaluation, and control.

Singh et al. add structural evidence by using diffusion tensor imaging and diffusion kurtosis imaging to compare brain microstructure in ME/CFS, long COVID, and healthy controls. ME/CFS was associated with microstructural alterations in regions including the cingulum, supplementary motor areas, and parts of the corpus callosum. Long COVID showed alterations in regions including the fusiform gyrus, precentral gyrus, and major white-matter tracts. Direct comparison between ME/CFS and long COVID identified a group difference in the left corona radiata. By applying complementary diffusion-based imaging approaches, the study supports the view that ME/CFS and long COVID share clinical features but may also show partly distinct neurobiological patterns. The partly distinct imaging patterns may reflect SARS-CoV-2-specific effects added to mechanisms shared across post-infectious syndromes. In long COVID, endothelial injury, blood–brain barrier disruption, and inflammation may alter glial cells and myelin, contributing to radial white-matter abnormalities. In longstanding ME/CFS, axial and callosal changes may reflect different tissue involvement or more chronic remodeling. Both conditions affected networks related to movement and cognitive effort, with ME/CFS involving movement planning and emotional integration, and long COVID involving motor pathways and visual-social processing.

Autonomic and regulatory dysfunction is another major theme. Miwa reviews orthostatic intolerance in ME/CFS and argues that cardiovascular abnormalities and disequilibrium should both be considered. The review discusses the “small heart” phenotype, reduced left ventricular size, low cardiac output, and downregulation of volume-regulating systems such as the renin–aldosterone and antidiuretic hormone pathways. It also emphasizes that postural instability or disequilibrium, possibly related to central vestibular dysfunction, may contribute to orthostatic symptoms. Rather than replacing cardiovascular explanations, this perspective broadens them by suggesting that orthostatic intolerance in ME/CFS may arise from combined circulatory and postural-control abnormalities (Miwa).

Mancini et al. provide a complementary exercise physiology perspective. Using serial 2-day cardiopulmonary exercise testing, they found that dysfunctional breathing and hyperventilation were common in patients with ME/CFS. These abnormalities occurred despite peak oxygen consumption being comparable to sedentary controls. This finding suggests that exercise intolerance in some patients may reflect abnormal ventilatory regulation rather than reduced aerobic capacity alone. The study also raises the possibility that breathing-pattern assessment may identify a treatable component in a subset of patients (Mancini et al.).

Three further contributions address chronic fatigue from the perspectives of population function, long-term prognosis, and potential intervention. Ma et al. studied chronic fatigue in a large working-age population rather than in a clinically diagnosed ME/CFS cohort. They found that chronic fatigue was associated with lower multidimensional physical fitness and slower choice reaction time. These findings should be interpreted as evidence of functional impairment along a fatigue continuum, not as proof of a disease-specific mechanism. Still, the study is useful because it links fatigue symptoms with measurable cognitive–motor slowing and reduced physical capacity in a large community sample.

Jason et al. provide important longitudinal evidence from a prospective cohort followed after infectious mononucleosis. Their 7-year follow-up showed that illness severity at 6 months strongly predicted long-term persistence. Most participants classified as having severe ME/CFS at 6 months continued to meet diagnostic criteria 7 years later, whereas those with moderate or lingering symptoms had higher recovery rates. These findings strengthen the view that early post-infectious severity may identify a subgroup at greater risk for chronic illness. They are also consistent with emerging evidence that EBV can persist in autoreactive B cells and reprogram them into inflammatory, disease-promoting antigen-presenting cells. If a similar mechanism is confirmed in ME/CFS or Long COVID, it could support therapeutic strategies aimed at selectively depleting, silencing, or reprogramming these pathogenic B-cell populations (1).

Friedberg and LeBaron review molecular hydrogen, mainly administered as hydrogen-rich water, as a possible adjunctive treatment for ME/CFS. Their rationale is based on the antioxidant, anti-inflammatory, and mitochondrial effects of molecular hydrogen, which overlap with biological disturbances proposed in ME/CFS. The clinical evidence remains preliminary, with small studies and limited objective biomarker data. Nevertheless, the review identifies molecular hydrogen as a low-burden intervention that deserves further testing in larger, controlled trials with biological and functional outcomes.

**Figure 1:**
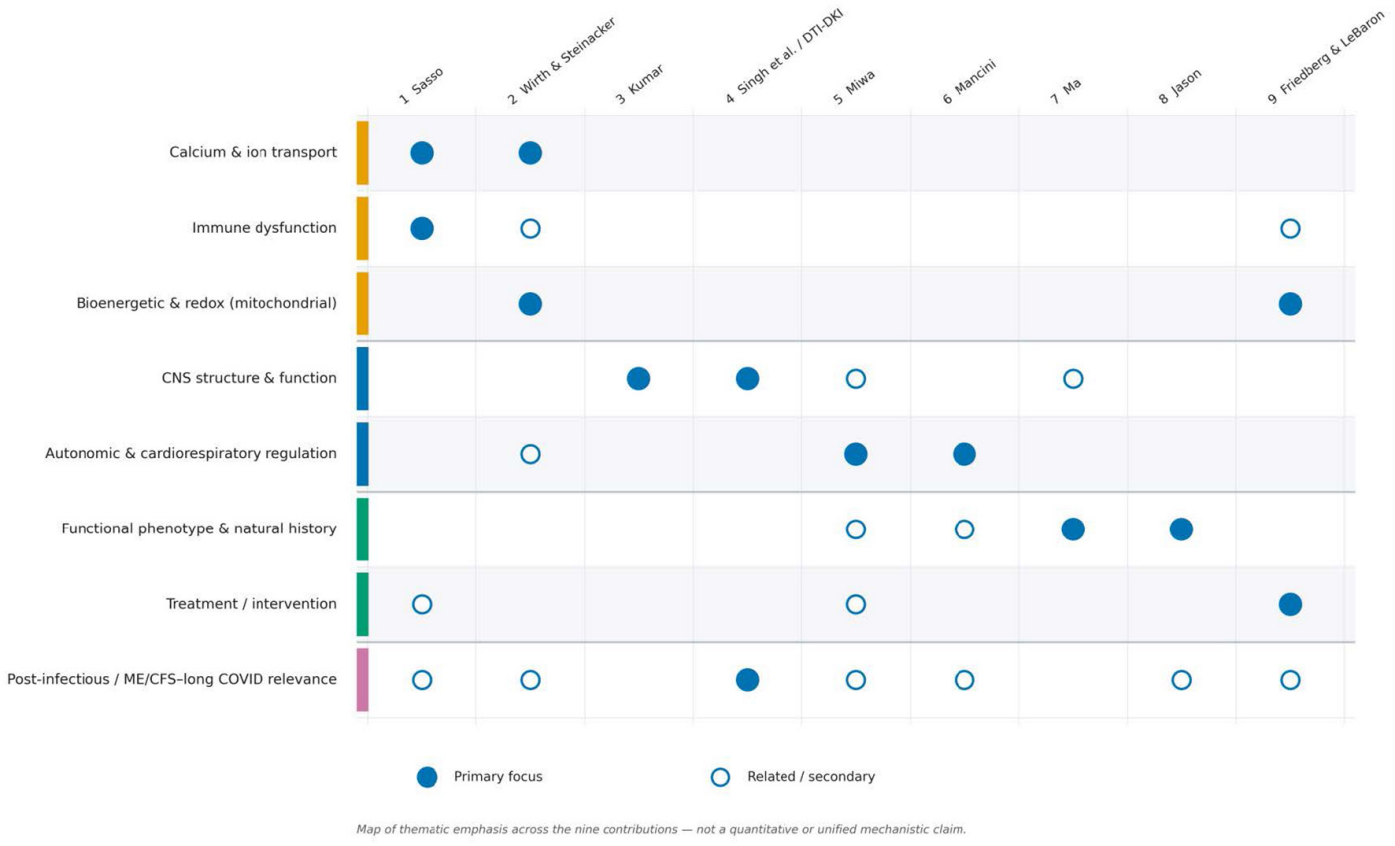
Thematic map of the nine contributions to the Research Topic across recurring mechanistic and clinical dimensions. Rows denote dimensions grouped by family (molecular/cellular—calcium and ion transport, immune dysfunction, bioenergetic and redox; neural—CNS structure and function, autonomic and cardiorespiratory regulation; clinical—functional phenotype and natural history, treatment; and a cross-cutting post-viral axis shared with long COVID), indicated by the colored tabs and separators. Columns denote the nine contributions (numbered as in the text). Filled circles mark a dimension that is a primary focus of a study; open circles mark a related or secondary contribution. The figure summarizes thematic emphasis only and is not a quantitative or mechanistic claim; immune and metabolic–oxidative pathways in particular recur across the underlying studies but are characterized only partially within this Research Topic.

Although these topics may appear distinct when examined individually as shown in Figure 1: ion-channel and calcium regulation, central nervous system structure and processing, autonomic and ventilatory control, muscle electrophysiology, and bioenergetic or oxidative stress pathways, together they suggest a broader, potentially unified multisystem framework. Mechanisms such as channelopathy and pathogen-driven autoimmunity, including possible EBV-associated changes in autoreactive B cells, may affect both peripheral tissues and the central nervous system, either independently or in combination. Their downstream effects on neural, cardiovascular, respiratory, immune, and muscular function could help explain the wide range of symptoms observed over the course of ME/CFS, as well as the substantial heterogeneity among individual patients and across study cohorts.

The evidence, however, remains provisional. Case definitions differ across studies, some contributions are hypothesis-driven or observational, and several outcomes rely on self-report. Candidate biomarkers have not yet been adequately tested for specificity against fibromyalgia, depression, autoimmune disease, and other fatiguing conditions. Severely ill patients also remain underrepresented. These limitations define the next steps clearly: determine the clinical specificity of promising assays such as TRPM3 testing; examine ion-channel, immune, bioenergetic, autonomic, respiratory, and central nervous system abnormalities within the same patients; identify how these mechanisms interact across different disease stages and severity groups; and test mechanism-based interventions in adequately powered, severity-stratified trials.

A distinctive strength of this Research Topic is its engagement with both ME/CFS and long COVID. Although these conditions should not be conflated, their substantial clinical and biological overlap provides an unprecedented research opportunity. The epidemiologic scale, often identifiable infectious onset, and increased scientific attention and resources surrounding long COVID create a large-scale comparative framework that may accelerate discovery in both conditions. Direct, phenotype-matched comparisons could help distinguish mechanisms shared across post-infectious illnesses from those associated with specific pathogens, disease stages, or patient subgroups. Such work is essential for moving beyond symptom-defined syndromes toward biologically grounded diagnosis, patient stratification, and targeted treatment.
